# HeMoDU: High-Efficiency Multi-Object Detection Algorithm for Unmanned Aerial Vehicles on Urban Roads

**DOI:** 10.3390/s24134045

**Published:** 2024-06-21

**Authors:** Hanyi Shi, Ningzhi Wang, Xinyao Xu, Yue Qian, Lingbin Zeng, Yi Zhu

**Affiliations:** 1 Army Engineering University of PLA (AEU), Nanjing 210007, China; zhuyi73@126.com; 2 Anhui University (AHU), Hefei 230601, China; sporange3187@gmail.com; 3 National University of Defense Technology (NUDT), Changsha 410073, China; xuxinyao0601@163.com (X.X.); zenglingbin16@nudt.edu.cn (L.Z.)

**Keywords:** UAV applications, object detection, computer vision, deep learning

## Abstract

Unmanned aerial vehicle (UAV)-based object detection methods are widely used in traffic detection due to their high flexibility and extensive coverage. In recent years, with the increasing complexity of the urban road environment, UAV object detection algorithms based on deep learning have gradually become a research hotspot. However, how to further improve algorithmic efficiency in response to the numerous and rapidly changing road elements, and thus achieve high-speed and accurate road object detection, remains a challenging issue. Given this context, this paper proposes the high-efficiency multi-object detection algorithm for UAVs (HeMoDU). HeMoDU reconstructs a state-of-the-art, deep-learning-based object detection model and optimizes several aspects to improve computational efficiency and detection accuracy. To validate the performance of HeMoDU in urban road environments, this paper uses the public urban road datasets VisDrone2019 and UA-DETRAC for evaluation. The experimental results show that the HeMoDU model effectively improves the speed and accuracy of UAV object detection.

## 1. Introduction

As global urbanization accelerates, traffic conflicts are becoming increasingly prominent. Road congestion and frequent accidents have become persistent problems plaguing urban development. To promote the rational use of road resources and improve traffic efficiency, it is crucial to explore an effective traffic detection method that can accurately obtain road traffic status. Currently, traffic detection equipment is primarily categorized into fixed traffic detectors and mobile traffic detection devices. Due to limitations in the number of installations and discontinuous data recording, it is challenging for fixed traffic detectors, such as microwaves, radar, and surveillance cameras, to accurately assess traffic conditions on continuous road sections. In contrast, mobile detection devices, e.g., satellite systems [1], floating cars, and UAVs, have the advantage of mobility. Among these, UAV-based detection devices stand out for their strong flexibility and high sampling rate and resolution, allowing for real-time, continuous, and comprehensive monitoring of road traffic information in complex urban environments. This, in turn, can provide effective assistance for important events such as traffic incident management, emergencies, and rescue operations.

The used object detection algorithm is integral to UAV road traffic detection applications. Traditional object detection algorithms suffer from low extraction and classification efficiency, poor adaptability to complex scenes, and high complexity requiring manual parameter tuning, making them unsuitable for meeting the performance requirements for object detection in dynamic and complex road environments. Deep-learning-based object detection algorithms inherit the advantages of deep learning algorithms, which have high precision and speed, enabling rapid and accurate image processing in complex large-scale environments. As such, they have gradually become the mainstream approach in the UAV object detection field.

In recent years, for different detection scenarios and tasks, researchers at home and abroad have proposed various neural network algorithms for object detection, such as DMNet [2], MPFPN [3], NAS-FPN [4], ClusDET [5], and the YOLO [6,7,8] series models. Among them, YOLOv8 is currently the state-of-the-art object detection model, as it can meet real-time requirements while maintaining high precision. However, the efficiency of the YOLOv8 object detection algorithm still needs improvement. On the one hand, it is necessary to improve the detection accuracy of the model to meet the requirements of complex road environments. On the other hand, the model’s training time and computational resource requirements need to be optimized for application in computationally limited UAV video sensors.

Given this context, this paper presents a deep-learning-based high-efficiency multi-object detection algorithm for UAVs (HeMoDU), employing several innovative techniques to achieve high-speed, accurate, UAV-based object detection for urban roads.

The contributions of this paper are as follows:

- This paper proposes a novel detection model based on YOLOv8 for the road traffic detection of UAVs. The model is named HeMoDU and significantly improves the object detection performance of YOLOv8.
- The visual state space concept from the VMamba model is introduced to HeMoDU using a 2D selective scan mechanism to obtain a global receptive field of the image at the cost of linear complexity, thereby extracting deeper image features.
- HeMoDU uses the VoV-GSCSP module from Slim-Neck to optimize the backbone and GSConv mixed convolution to fully utilize inter-channel connections at a small computational cost, which effectively improves the accuracy of small object detection and reduces model redundancy through effective processing of combined high-level features.
- HeMoDU employs the Programmable Gradient Information (PGI) framework to further enhance the model’s inference efficiency.

We conducted extensive experiments, including an ablation study and comparative experiments with state-of-the-art methods, to validate the efficiency of HeMoDU, and the experimental results show its superiority.

The organizational structure of this paper is as follows: Section 2 introduces the research background, analyzes the application prospects of UAVs in traffic detection, and outlines the development status of traffic detection algorithms based on deep learning; Section 3 describes the HeMoDU model; Section 4 details the experimental evaluations of the HeMoDU model; Section 5 discusses the limitations of the work and future research trends; and Section 6 concludes the paper.

## 2. Background

Unmanned aerial vehicles (UAVs) are highly integrated, easy-to-operate, unpiloted flying vehicles capable of carrying various sensors, offering high maneuverability and flexibility. Equipping UAV systems with video sensors for the purpose of traffic detection has advantages, such as a high collection efficiency, a wide detection range, and minimal impact on traffic, greatly improving traffic detection efficiency and reducing safety risks. At the same time, with the improved performance of computing chips onboard UAVs, the computational capability of UAVs has rapidly increased, allowing deep learning algorithms to be deployed on these devices and presenting new opportunities for enhancing the efficiency of UAV-based traffic detection.

This section provides an overview of traditional UAV traffic detection methods and deep-learning-based traffic detection algorithms.

### 2.1. Traditional UAV-Based Traffic Detection Methods

In traffic detection, UAVs can be used as devices for environmental perception and information acquisition, obtaining road and vehicle data through components such as high-definition video sensors, infrared detection sensors, and communication devices. Based on the collected data, various road traffic state parameters such as vehicle trajectories, speeds, road traffic density, and traffic volume can be extracted, thereby providing data support for traffic decision-making and management.

Scholars at home and abroad have fully leveraged the advantages of UAVs, such as their strong maneuverability and wide field of view, achieving rich results in UAV-based traffic detection. Hinz et al. proposed a vehicle detection method based on collecting monocular aerial images using UAVs, constructing hierarchical 3D models, and computing results through an image-matching model and evaluation algorithm [9]. Fuqiang Liu et al. proposed a framework for vehicle detection and multi-target tracking algorithms based on UAV data, extracting traffic flow information by tracking vehicles through the construction of specific data structures [10]. Jing Dong et al. introduced a real-time object detection tracking algorithm that enhanced the real-time performance of moving object detection in road videos captured by UAVs [11]. Imran Saleemi et al. presented an algorithm framework capable of tracking thousands of vehicle targets in high-resolution, low-frame-rate videos obtained from multiple cameras and introduced a weighted hypothesis measure to optimize for issues like occlusion and false detections [12]. Xin Zhang et al. developed an algorithm to track vehicles from UAV-acquired videos and extract their speeds, calculating vehicle speeds based on vehicle motion trajectories and affine models [13]. Kim et al. proposed a road traffic monitoring method based on UAV posture control, which not only enables vehicle detection and analysis of abnormal traffic states but also allows for preliminary classification of sudden and catastrophic traffic conditions. The method enhances the efficiency of UAV application in road traffic detection by selecting corresponding controls for the UAV based on detected results [14].

### 2.2. Deep-Learning-Based Traffic Detection Methods

With the widespread application of deep learning in computer vision, an increasing number of traffic detection algorithms based on deep learning have been proposed, such as convolutional neural networks (CNNs) [15], recurrent neural networks (RNNs) [16], and semantic segmentation algorithms [17]. Compared to traditional detection methods, deep-learning-based object detection algorithms can automatically learn feature representations from massive amounts of data and possess high generalization and adaptability.

In recent years, scholars at home and abroad have continuously improved vehicle detection models based on deep learning object detection networks, enhancing detection accuracy and achieving numerous research results. Among these, the YOLO (You Only Look Once) object detection algorithm proposed by Redmon et al. in 2016 has gone through several generations of updates and has gradually become a mainstream framework for object detection. The initial YOLO algorithm standardized the input image size and used a deep convolutional neural network along with model confidence to determine the target’s position, but it performed poorly at detecting small objects and had significant localization errors. Redmon et al. then introduced the YOLO9000 algorithm [18], which achieved a better balance between detection speed and precision by employing a novel multi-scale training approach. In 2018, the YOLOv3 algorithm [19] was proposed. The YOLOv3 algorithm incorporates FPN into YOLOv2 and is trained using the Darknet-53 network, tripling the detection speed while maintaining SSD-like precision. In 2020, Bochkovskiy et al. presented the YOLOv4 algorithm [20], combining CSPDarknet-53 feature extraction and SPP networks, introducing Mosaic for data augmentation, and enhancing speed, accuracy, and generalization capabilities. Two months after YOLOv4, Ultralytics released the YOLOv5 algorithm [21], which adopted Mosaic data augmentation [22], adaptive anchor box computation, and adaptive image scaling at the input end. Its framework includes a focus structure, a CSP-based backbone, and an FPN-based neck, showing greater stability against gradient explosion. In 2022, Li et al. proposed the anchor-box-free YOLOv6 algorithm [23]. They designed a re-parameterizable and more efficient backbone network called EfficientRep and a Rep-PAN neck layer based on RepVGGstyle during the feature extraction stage, further improving detection accuracy. That same year, Alexey et al. designed YOLOv7 [24], which employs new data augmentation techniques like Mixup, Copy-Paste, and Paste-In, combining SPPCPC layers, BConv layers, MPConv layers, and RepVGG block layers for feature map prediction and enhancing detection precision for mobile GPU devices. In 2022, Lei Yang et al. [25] proposed an improved, high-performance image detection model called RS-YOLOX, which improved the detection of small targets by using ECA and ASFF. In 2023, they further improved YOLOX-Nano’s performance by implementing several lightweight improvement strategies to increase the accuracy and speed of the model [26]. In addition, Chun Liu et al. [27] proposed an innovative algorithm incorporating an adaptive multi-scale feature enhancement and fusion module (ASEM), which enhances object detection through sophisticated multi-scale feature fusion.

UAVs have unique advantages in the field of traffic detection, and deep learning algorithms exhibit high adaptability and strong generalization capabilities. In the future, deep-learning-based UAV traffic detection algorithms are expected to play a more significant role in practical applications. In light of this, deep-learning-based UAV object detection was researched in this study, with the aim of designing and proposing a high-speed, precise UAV road object detection algorithm suitable for urban road environments.

## 3. Proposed Method

In the field of view of UAVs, moving targets in actual urban road scenes exhibit significant multi-scale and high-density characteristics, making object detection highly challenging. To achieve effective detection of urban road targets, this paper reconstructs the state-of-the-art YOLOv8 model, makes corresponding improvements to suit the multi-scale characteristics of urban road targets, and proposes the high-efficiency multi-object detection algorithm for UAVs (HeMoDU), which effectively enhances the model’s feature extraction capabilities and object detection performance.

**Figure 1:**
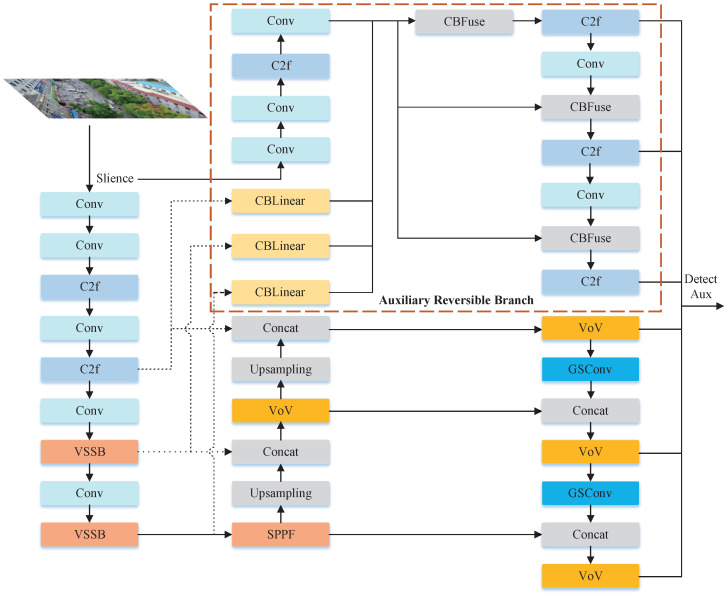
Basic structure of the proposed HeMoDU model, where VSSB indicates the VSS block and VoV is the VoV-GSCSP.

The basic architecture of HeMoDU is shown in Figure 1. The optimizations we made are as follows: First, we replaced two deep-level CSP bottleneck layers with two convolutions (C2f) in the backbone part of YOLOv8 with the VMamba basic module VSS Block (VSSB) to achieve sufficient deep feature extraction. Then, we introduced GSConv to speed up the computation of the predicted image in the CNN model and wrapped it into the VoV-GSCSP (VoV) module to further integrate the corresponding features. Finally, we adopted Programmable Gradient Information (PGI) to address the substantial information loss occurring during the feature extraction and spatial transformation processes layer-by-layer.

In this section, we first introduce our method in three parts, i.e., VSS Block, VoV-GSCSP, and PGI, and then we present the loss function we leveraged.

### 3.1. VSS Block

**Figure 2:**
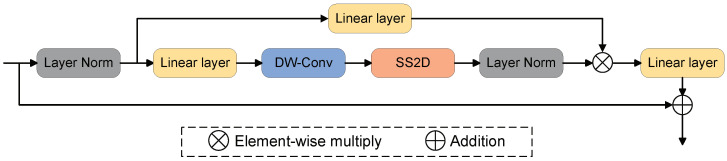
Framework of the VSS Block, which contributes as the main part of HeMoDU. SS2D is the core function.

The basic VSS block module from the latest VMamba architecture is introduced to HeMoDU. The VSS block achieves a global receptive field with linear complexity through its 2D selective scan mechanism, thus enabling improved capture of deep features in images, as shown in Figure 2.

This module divides the input into two branches after the feature map undergoes layer normalization. In the first branch, the feature map goes through a linear transformation then multiplies with a weight matrix followed by the activation function SiLU. In the second branch, the input also passes through a linear layer and the corresponding activation function and then uses depthwise separable convolution for shallow feature extraction with a small number of parameters. After this, it enters the 2D selective scan (SS2D) module for further deep feature extraction. Subsequently, layer normalization is used to normalize the thoroughly extracted features, which are merged with the first branch’s output through element-wise multiplication to form a deep feature representation that includes shallow information. Finally, a linear layer is used to mix the features, and the output of this layer is combined with residual connections to form the output of the VSS module.

**Figure 3:**
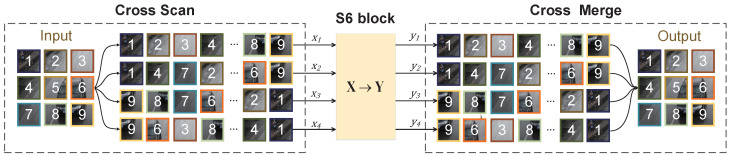
Illustration of the SS2D operation process.

SS2D consists of three parts: the scan expansion operation, the S6 module, and the scan merge operation. As shown in Figure 3, the image’s scanned blocks are expanded into four sequences, each consisting of several small-sized images obtained from different scan divisions. The scanning continues in four directions, allowing the central pixel to integrate information from other pixels in different directions. After scanning, the formed sequences are processed by the S6 module for feature extraction, ensuring that information from all directions is thoroughly scanned to capture different features. Then, the scan merge operation sums and merges the sequences from the four directions.

The function of the S6 module is detailed as follows:

(1) $$\begin{array}{cc} \bar{A} & =e^{\Delta A} \end{array}$$

(2) $$\begin{array}{cc} \bar{B} & ={\left(\Delta A\right)}^{-1}\left(e^{\Delta A}-I\right)\cdot \Delta B \end{array}$$

(3) $$\begin{array}{cc} h_{t} & =\bar{A}h_{t-1}+\bar{B}X_{t} \end{array}$$

(4) $$\begin{array}{cc} Y_{t} & =Ch_{t}+DX_{t} \end{array}$$

where $X\in R^{batch\;size\times token\;length\times dimension}$ is the input, and Y is the output, which has the same dimensions as X. A, B, and Δ are the transformation results from X via three fully connected layers, respectively. C and D are learnable matrices. I is the identity matrix with the same size as ΔA. $h_{t}$ represents the hidden state at time *t*.

### 3.2. VoV-GSCSP

To ensure the computational efficiency of the CNN model, the images fed into the CNN must undergo a similar transformation process in the backbone, i.e., progressively transferring spatial information into channels. Each time the spatial dimensions (width and height) of the feature map are compressed and the channels are expanded, some of the semantic information will be lost. Dense convolution computations preserve the hidden connections between each channel to the greatest extent, whereas sparse convolutions such as DW-Conv completely sever these connections, making it difficult for models that use a large number of depthwise separable convolutions to achieve the desired detection accuracy [28].

However, in actual urban road scenarios, numerous small targets require the model’s small object detection capabilities to be enhanced while ensuring computational efficiency. Slim-Neck utilizes GSConv mixed convolution to fully exploit the connections between channels, processing concatenated feature maps at stages where the channel dimension is sufficiently large and the spatial dimension is sufficiently small, thereby reducing the model’s number of network layers and amount of redundant information. This improves the accuracy of small object detection with a smaller number of parameters. Therefore, GSConv is introduced to HeMoDU, passing the input through regular convolution first, then performing depthwise convolution, and finally concatenating the two results. A shuffle operation is then performed, intertwining the channels corresponding to the previous two convolution results, making the depthwise convolution output more similar to the regular convolution, thus addressing the issue of low accuracy.

**Figure 4:**
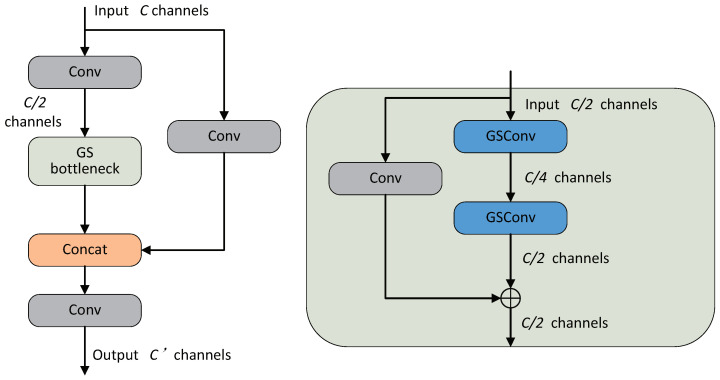
Structure of the VoV-GSCSP.

In HeMoDU, GSConv is applied within the VoV-GSCSP module, as shown in Figure 4. HeMoDU uses a one-shot aggregation strategy to design the cross-stage partial network module, VoV-GSCSP. The input features are extracted through convolutional layers and separable convolutional layers then concatenated with the information from the residual connection path that has been convolved to change the number of channels. In this way, HeMoDU preserves the hidden connections between channels and promotes cross-channel information exchange, thus maintaining sufficient accuracy while reducing computational and network structural complexity.

### 3.3. Programmable Gradient Information

**Figure 5:**
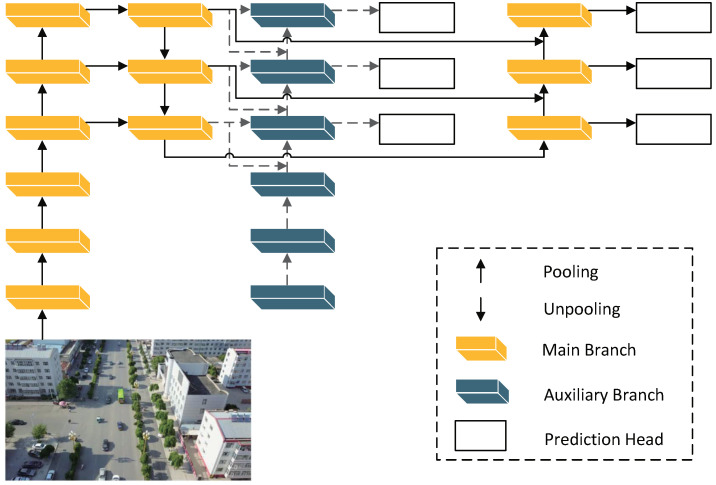
Structure of the PGI module.

To further enhance the model’s capability for extracting deep image features, this paper introduces the YOLOv9’s Programmable Gradient Information (PGI) [29] framework into HeMoDU, whose basic structure is shown in Figure 5.

The core idea of PGI is to introduce auxiliary supervisory signals to provide additional gradient information for the backbone network, achieving correction and enhancement of the original gradients during model training, thereby effectively improving both object detection performance and training efficiency.

In HeMoDU, the backbone network is the fundamental object detection network, responsible for feature extraction and output results. To address the gradient vanishing problem faced by deep neural network models, HeMoDU employs an auxiliary invertible branch that runs parallel to the backbone network. The auxiliary invertible branch provides additional gradient information for the backbone network without increasing the inference cost. During computation, HeMoDU can blend shallow features into deep features through invertible operations such as addition and multiplication to alleviate the information bottleneck issue. In addition, during the backpropagation of gradients, it directly channels gradients to the shallow layers through invertible operations, effectively preventing the gradient disappearance problem caused by deep neural network structures.

Furthermore, we designed a multi-level auxiliary information mechanism for the framework to enhance HeMoDU’s ability to extract effective multi-scale features. This mechanism introduces auxiliary supervisory signals constructed from the loss function at different levels of the invertible branch, assisting the main model in better learning multi-scale features. In addition, it controls the process of gradient flow to the backbone network by adjusting the supervisory weights at different levels, thus achieving “programmable” gradients.

Since the PGI only uses the main branch during inference, it does not generate additional inference time or computational resource consumption during the inference process. Therefore, without increasing inference costs, it effectively improves the model’s detection performance for multi-scale objects.

### 3.4. Loss Function

The loss function used by the proposed model consists of a classification loss and a frame regression loss. The main body of the classification loss is BCEWithLogitsLoss, which is a mixture of a sigmoid activation function and a binary cross-entropy loss function, and its mathematical expression is as follows:

(5) BCEWithLogitsLoss(x,y)=BCE(sigmod(x),y)=−ylog(σ(x))−(1−y)log(1−σ(x))

where *x* is the output of the model (logits), *y* is the real label (0 or 1), σ(·) represents the sigmoid activation function, and BCE(·,·) represents the two-category cross-entropy loss function. BCEWithLogitsLoss(x,y) here is actually equivalent to BCE(p,y), where p=σ(x) is the predicted probability value obtained by the sigmoid activation function.

We sum the binary cross-entropy loss of each target and then take the average to obtain the final classification loss. The loss of border regression is mainly composed of CIoU loss and DFL loss. CIoU loss is an improved version of IOU, which considers the complete intersection between target BOX and introduces correction information to measure the similarity between target BOX more accurately; it also enables the model to better understand the exact position and shape of target BOX during training. The formula for CIoU is defined as follows:

(6) $$\begin{array}{cc} CIoU & =IoU-(\frac{d^{2}}{c^{2}}+\alpha \nu ) \end{array}$$

(7) $$\begin{array}{cc} \nu & =\frac{4}{\Pi }{\left(arctan\frac{\omega^{gt}}{h^{gt}}-arctan\frac{\omega }{h}\right)}^{2} \end{array}$$

(8) $$\begin{array}{cc} \alpha & =\frac{\nu }{(1-IoU)+\nu } \end{array}$$

where ν calculates the similarity between the anchor frame and the target frame and α is the weight function. From the formula given by α, it can be seen that the optimization method of the loss function is to increase the overlapping area between the real frame and the target frame. The CIoU loss is defined by the following formula:

(9) $$L_{CIoU}=1-IoU+\left(\frac{d^{2}}{c^{2}}+\alpha \nu \right)$$

Therefore, the advantage of $L_{CIoU}$ is that it can directly minimize the distance between the target frame and the real frame, which makes the convergence speed and the effect of the loss function better.

DFL loss was put forward as generalized focal loss, which is used to make a network quickly focus on the value near the label and make the probability density at the label as large as possible. The idea is to use the cross-entropy function to optimize the probability of the left and right positions near the label *y* so that the network distribution is focused near the label value. The DFL function is defined as follows:

(10) $$DFL\left(S_{i},S_{i+1}\right)=-\left(\left(y_{i+1}-y\right)log\left(S_{i}\right)+\left(y-y_{i}\right)log\left(S_{i+1}\right)\right)$$

where S(·) is the softmax function. DFL expands the probability around the truth value *y*.

Our loss is obtained by multiplying the above components by the corresponding coefficients $\lambda_{BCE}$, $\lambda_{BOX}$, and $\lambda_{DFL}$. Here, we take $\lambda_{BCE}$ to be 0.5, $\lambda_{BOX}$ to be 7.5, and $\lambda_{DFL}$ to be 1.5.

## 4. Evaluation

We utilized the publicly available urban road datasets VisDrone2019 and UA-DETRAC to validate the effectiveness of our method. All experiments were based on Python 3.7 and PyTorch 1.11 software and Intel i9-12700 and Nvidia 3090 hardware.

### 4.1. Dataset Introduction

VisDrone2019 and UA-DETRAC are two widely used public datasets for urban road object detection and tracking. A detailed introduction to these two datasets is presented below.

(1) VisDrone2019 Dataset

**Figure 6:**
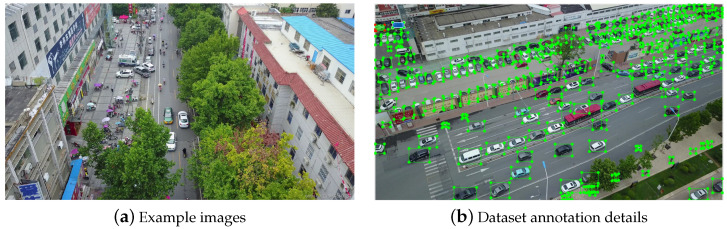
Sample of the VisDrone2019 dataset.

The VisDrone2019 dataset [30] was collected and developed by the AISKYEYE team from the Machine Learning and Data Mining Laboratory at Tianjin University. The images and video clips in this dataset were captured by various UAV cameras, spanning thousands of kilometers across 14 cities, and include different geographical environments such as urban and rural areas, different objects such as pedestrians and vehicles, and different densities such as sparse and crowded, totaling 10,209 samples. Among them, 6471 samples were used for training, 548 for validation, and 3190 for testing, with more than 2.6 million target boxes manually annotated. Figure 6 shows some samples and annotations from this dataset. The images contain rich content, including a large number of targets with multi-scale and uneven distribution characteristics. This dataset categorizes these targets into 11 classes: pedestrian, people, bicycle, car, van, truck, tricycle, awning-tricycle, bus, motor, and others. This dataset can be used for multi-object detection tasks in urban road scenarios.

(2) UA-DETRAC Dataset

The UA-DETRAC dataset is a widely used, large-scale, open-source dataset for vehicle detection and tracking [31]. This dataset serves as a challenging benchmark for real-world multi-object detection and tracking models. The data primarily consist of high-definition video surveillance footage from 24 roads in Beijing and Tianjin, recorded at 25 frames per second with a resolution of 960 × 540 pixels. The data include scenarios across four weather conditions: sunny, rainy, daytime, and nighttime. Researchers have categorized the vehicles in the dataset into four classes: car, bus, van, and others. Based on this categorization, over 140,000 frames and 8250 vehicles were manually annotated, totaling 1.21 million object-bounding boxes. The training set contains approximately 82,085 images, while the test set contains about 56,167 images.

**Figure 7:**
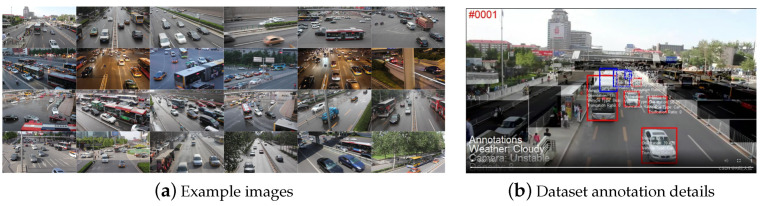
Sample of the UA-DETRAC dataset.

Figure 7 illustrates typical examples from the UA-DETRAC dataset and an annotated frame. Red bounding boxes indicate fully visible targets, while blue and pink boxes represent partially occluded targets. The vehicle’s ID, direction of travel, type, and truncation rate are displayed within the bounding box. Additionally, the weather conditions, camera status, and vehicle density are shown in the bottom left corner of each frame.

### 4.2. Analysis Experiments

Firstly, the effectiveness of the method proposed in this paper on the UA-DETRAC dataset was verified through ablation experiments. In this experiment, under all experimental settings, the models were trained with the SGD optimizer using a mini-batch strategy and a batch size of 16 for 150 epochs, with a learning rate of 0.01; Mosaic [32] was used for data augmentation during training.

The experiment employed metrics commonly used in object detection tasks, such as precision, recall, mean average precision (mAP) at an IoU threshold of 0.5 (mAP50), and mAP between IoU thresholds of 0.5 and 0.95 (mAP50-95), to test the effectiveness of the proposed model [33]. Table 1 shows the specific test results. In the table, “baseline” refers to the YOLOv8 model [34], while vss, vov, and pgi represent the VSS module, VoV-GSCSP module, and the Programmable Gradient Information (PGI) framework in the model, respectively. As shown in Table 1, each module adopted by our method effectively improved the performance of YOLOv8 in terms of the detection accuracy, mAP50, and mAP50-95 metrics. In object detection tasks, there is often a trade-off between precision and recall. Since positive samples such as vehicles and pedestrians constitute a small proportion of all monitored samples in real traffic environments, the detection model needs to accurately detect and recognize these positive samples. Considering the actual task requirements, this method places more emphasis on the detection accuracy of the model, which leads to limited improvement in recall performance. However, the improved model surpasses the baseline YOLOv8 model in all metrics, thus proving the effectiveness of the proposed method.

We also conducted another ablation study to evaluate the Param, GFLOPs, and FPS of HeMoDU, as shown in Table 2. Firstly, the number of parameters revealed that we had successfully compressed the Param, leading to a reduction in both the model’s size and its spatial complexity. Concurrently, we augmented the global floating-point operations, enabling the model to assimilate more data and enhance its performance. Nonetheless, integrating more sophisticated modules rendered the inference procedure potentially more intricate than the baseline, culminating in a diminished FPS value. Furthermore, it was observed that the Param and GFLOPs for baseline+pgi were congruent with those of the baseline. This can be attributed to the fact that, during testing, the PGI framework employed in the training phase can be excised, facilitating performance enhancement without escalating the model’s intricacy. Consequently, this approach permits the preservation of the model’s dimensions while bolstering its frame rate.

### 4.3. Comparative Experiment

This section will further compare HeMoDU with current typical methods on the Visdrone2019 dataset and the UA-DETRAC dataset to verify its superior performance. We compare HeMoDU with YOLOv3 [19], YOLOv4 [20], Li et al. [35], DFE-Net [36], EM-YOLO [37], YOLOv8s [34], and EdgeYOLO [38] on the Visdrone2019 dataset and with YOLOv5s, YOLOv5-NAM [39], Peng et al. [40], and YOLOv8s on the UA-DETRAC dataset.

The specific detection results are shown in Table 3 and Table 4. Under most experimental settings, HeMoDU performs better than most of the compared methods in terms of accuracy, mAP-50, and mAP50-90 metrics. The experimental results indicate that, compared with the other methods, HeMoDU can maintain higher detection accuracy while possessing stronger generalization ability for tasks of varying difficulty, hence demonstrating greater applicability.

We further display the detection performance of HeMoDU on typical targets in traffic scenes, as shown in Table 5 and Table 6. In the Visdrone-2019 dataset, much fewer samples belong to the children’s tricycle category and the bicycle category compared to other data categories. Meanwhile, the sizes of these samples are much smaller as well. As the results show, the detection performance of the proposed HeMoDU is worse in the two categories than in the other categories. Although multi-label target recognition is still a highly challenging task, HeMoDU can still effectively detect main road targets such as cars, pedestrians, and buses.

**Figure 8:**
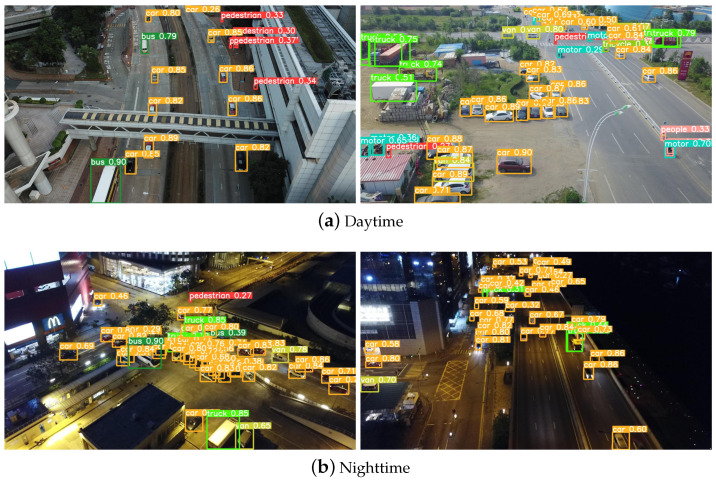
Visualization results on the VisDrone2019 dataset.

**Figure 9:**
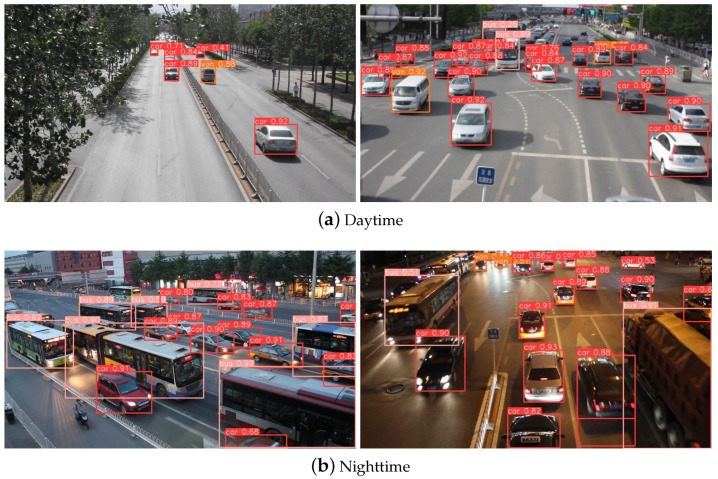
Visualization results on the UA-DETRAC dataset.

**Figure 10:**
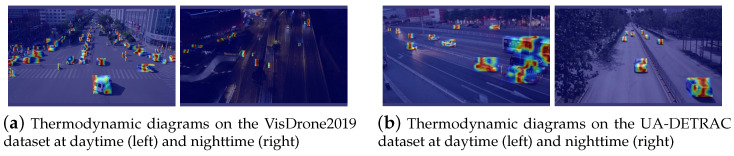
Thermodynamic diagrams on VisDrone2019 and UA-DETRAC datasets at different times.

Figure 8 and Figure 9 show the visualized detection results of the samples in the Visdrone2019 dataset and the UA-DETRAC dataset, respectively. The proposed HeMoDU can precisely classify objects in road scenes with both clear and dim lighting conditions. It is able to distinguish the pedestrian objects from the car objects in heavy traffic scenes as well, as evidenced by Figure 8a. As shown in Figure 9, the proposed HeMoDU model can also identify car objects of multiple sizes precisely, so the method performs well on the car and bus categories, as shown in Table 6. In addition, Figure 10 shows the corresponding thermodynamic diagrams. In both figures, HeMoDU can effectively focus on the features corresponding to the main targets. During the day, HeMoDU can accurately identify multi-scale targets, including bicycles, which enables the model to have a good detection effect on targets with different scale features. In addition, under the highly challenging nighttime scene, HeMoDU can still effectively recognize typical road targets such as cars. This demonstrates the capability of HeMoDU for object detection under complex conditions.

## 5. Future Research

The HeMoDU model proposed in this paper has demonstrated commendable efficiency and accuracy in recognizing urban road elements. However, to further optimize its performance, we believe it is essential to improve upon two key aspects:

(1) The model’s object detection capability in high-density or even saturated scenarios should be enhanced. The object detection task becomes particularly complex in environments where road elements are closely arranged. To boost the model’s detection capability in such challenging scenarios, there is a need to explore more advanced algorithms and techniques that can better handle occlusions, overlaps, and interference between targets. This may involve developing new sensor fusion strategies, refining existing deep learning architectures, or incorporating more sophisticated data augmentation techniques to enhance the model’s adaptability to complex scenes.

(2) The model’s ability to differentiate between morphologically similar mixed targets should be improved. In practical applications within urban road environments, it is common to encounter different targets with similar shapes or textures, necessitating a high degree of discriminative power in the object detection model. To augment the HeMoDU model’s capability in this regard, we plan to introduce more refined feature extraction mechanisms and design more targeted classifiers by integrating domain knowledge. Moreover, by adopting fine-grained classification and contrastive learning methods, we can further encourage the model to learn more nuanced discriminative features, thereby improving its accuracy in distinguishing similar targets.

In summary, through continuous research and improvement in the above-mentioned areas, we anticipate that the HeMoDU model will achieve new heights in the field of drone object detection and recognition, serving complex real-world application scenarios more effectively.

## 6. Conclusions

To enhance the efficiency of road object detection for UAVs, this paper proposes HeMoDU, which reconstructs the YOLOv8 module. Firstly, HeMoDU replaces two deep-level CSP BottleNeck layers with two convolutions (C2f) in the backbone part of YOLOv8 with the VMamba basic module VSS Block (VSSB) to achieve sufficient deep feature extraction. Then, GSConv is introduced to HeMoDU to accelerate the computation of prediction images in the convolutional neural network (CNN) and encapsulates it into the VoV-GSCSP module to further integrate the corresponding features. Finally, HeMoDU adopts Programmable Gradient Information (PGI) to address the issue of significant information loss during the layer-by-layer processes of feature extraction and spatial transformation. The experimental results show that the performance of HeMoDU on the Visdrone2019 and UA-DETRAC datasets surpasses that of the most advanced YOLO series models. In addition, HeMoDU can achieve high detection effectiveness in both daytime and nighttime scenarios. 

## Figures and Tables

**Table 1:** Performance of HeMoDU with different employed models on the UA-DETRAC dataset.

Model	P	R	mAP50	mAP50-95
Baseline	0.605	0.623	0.622	0.484
Baseline+vss	0.660	0.641	0.646	0.485
Baseline+vov	0.623	0.631	0.628	0.470
Baseline+pgi	0.673	0.627	0.651	0.486
HeMoDU ^1^	0.719	0.599	0.657	0.500

^1^ HeMoDU indicates Baseline+vss+vov+pgi.

**Table 2:** Performance of HeMoDU combined with different models on the UA-DETRAC dataset.

Model	Param	GFLOPs	FPS
Baseline	25.8 M	78.7	154.7
Baseline+vss	20.0 M	82.5	95.5
Baseline+vov	16.9 M	56.2	68.3
Baseline+pgi	25.8 M	78.7	169
HeMoDU	21.3 M	80.3	100.3

**Table 3:** Performance of the compared models on the Visdrone-2019 dataset.

Model	P	R	mAP50	mAP50-95
YOLOv3	0.530	0.436	0.419	0.233
YOLOv4	0.360	0.486	0.421	0.257
Li et al. [35]	0.592	0.431	0.44	0.254
DFE-Net	0.484	0.391	0.393	0.220
EM-YOLO	-	-	0.435	0.251
YOLOv8s	0.509	0.382	0.393	0.235
EdgeYOLO	0.502	0.436	0.448	0.262
HeMoDU	0.549	0.424	0.442	0.271

**Table 4:** Performance of the compared models on the UA-DETRAC dataset.

Model	P	R	mAP50	mAP50-95
YOLOv5s	0.583	0.492	0.503	0.325
YOLOv5-NAM	0.596	0.496	0.517	0.340
Peng et al. [40]	0.615	0.472	0.596	0.442
YOLOv8s	0.605	0.623	0.622	0.484
HeMoDU	0.719	0.599	0.657	0.500

**Table 5:** Object detection efficiency of HeMoDU on the Visdrone-2019 dataset.

Model	P	R	mAP50	mAP50-95
All	0.549	0.424	0.442	0.271
Pedestrian	0.593	0.430	0.479	0.228
People	0.604	0.311	0.373	0.150
Bicycle	0.326	0.189	0.170	0.078
Car	0.766	0.788	0.820	0.599
Van	0.576	0.484	0.501	0.360
Truck	0.580	0.401	0.421	0.287
Tricycle	0.459	0.355	0.342	0.195
Awning-tricycle	0.335	0.216	0.190	0.120
Bus	0.712	0.574	0.633	0.462
Motor	0.540	0.490	0.488	0.231

**Table 6:** Object detection efficiency of HeMoDU on the UA-DETRAC dataset.

Model	P	R	mAP50	mAP50-95
All	0.719	0.599	0.657	0.500
Car	0.743	0.720	0.771	0.576
Bus	0.722	0.779	0.791	0.609
Van	0.562	0.584	0.591	0.464
Others	0.666	0.424	0.451	0.294

## Data Availability

Publicly available datasets used in this paper can be accessed at https://github.com/VisDrone/VisDrone-Dataset (Visdrone2019, accessed on 13 May 2024) and http://detrac-db.rit.albany.edu/ (UA-DETRAC, accessed on 13 May 2024).
